# Rostral and caudal prefrontal contribution to creativity: a meta-analysis of functional imaging data

**DOI:** 10.3389/fnhum.2013.00465

**Published:** 2013-08-14

**Authors:** Gil Gonen-Yaacovi, Leonardo Cruz de Souza, Richard Levy, Marika Urbanski, Goulven Josse, Emmanuelle Volle

**Affiliations:** ^1^Department of Psychology, Ben-Gurion University of the NegevBeer-Sheva, Israel; ^2^Centre de Recherche de l'Institut du Cerveau et de la Moelle épinière, Université Pierre et Marie Curie-Paris 6, UMRS 975Paris, France; ^3^Institut National de la Santé et de la Recherche Médicale, U 975Paris, France; ^4^Centre National de la Recherche Scientifique, UMR 7225Paris, France; ^5^AP-HP, Service de Neurologie, Hôpital Saint-AntoineParis, France; ^6^Service de Médecine et Réadaptation, Hôpitaux de Saint-MauriceSaint-Maurice, France

**Keywords:** creativity, meta-analysis, divergent thinking, insight problem solving, creative thinking, functional imaging, semantic associations, originality

## Abstract

Creativity is of central importance for human civilization, yet its neurocognitive bases are poorly understood. The aim of the present study was to integrate existing functional imaging data by using the meta-analysis approach. We reviewed 34 functional imaging studies that reported activation foci during tasks assumed to engage creative thinking in healthy adults. A coordinate-based meta-analysis using Activation Likelihood Estimation (ALE) first showed a set of predominantly left-hemispheric regions shared by the various creativity tasks examined. These regions included the caudal lateral prefrontal cortex (PFC), the medial and lateral rostral PFC, and the inferior parietal and posterior temporal cortices. Further analyses showed that tasks involving the combination of remote information (combination tasks) activated more anterior areas of the lateral PFC than tasks involving the free generation of unusual responses (unusual generation tasks), although both types of tasks shared caudal prefrontal areas. In addition, verbal and non-verbal tasks involved the same regions in the left caudal prefrontal, temporal, and parietal areas, but also distinct domain-oriented areas. Taken together, these findings suggest that several frontal and parieto-temporal regions may support cognitive processes shared by diverse creativity tasks, and that some regions may be specialized for distinct types of processes. In particular, the lateral PFC appeared to be organized along a rostro-caudal axis, with rostral regions involved in combining ideas creatively and more posterior regions involved in freely generating novel ideas.

## Introduction

Everyone has their own idea of what creativity is. While the realm of artistic creation may be the first that comes to mind, creativity is obviously a cornerstone of many domains of human activity, including science (discovery), technology (invention), and economy (innovation). However, it is not restricted to extraordinary achievements. Finding new solutions to individual problems, achieving something novel, and thinking away from pre-established ideas are all common creative processes that take place in everyday life. According to this point of view, creativity results from a set of mental functions normally found in all humans, and can be studied experimentally. From a neuroscience perspective, creativity is defined as the ability to produce work that is both novel (original) and appropriate or useful (Sternberg and Lubart, 1999; Plucker and Makel, 2010; Runco and Jaeger, 2012). Although this definition may appear reductive or simplistic, it makes experimental testing possible by allowing to form hypotheses about the cognitive processes involved in creativity, and to examine their brain correlates. However, the brain substrates of creativity have been poorly studied. In the existing research, various tasks related to four main theoretical frameworks of creativity have been used: divergent thinking, insight problem solving, combination of remote semantic associations, and artistic creativity. These studies have led to diverse results, with no consensus yet in sight (Fink et al., 2007; Arden et al., 2010; Dietrich and Kanso, 2010; Sawyer, 2011). The present meta-analysis represents an attempt to clarify this small body of literature.

Divergent thinking tasks assess the ability to generate multiple solutions to an open-ended problem that does not have a right or wrong answer (Guilford, 1950). The products of divergent tasks are evaluated according to several criteria, mainly fluency (the quantity of relevant responses), flexibility (the number of different categories of responses), originality (the degree to which responses are uncommon), and elaboration (the degree of enrichment of responses). In a review of six functional imaging studies that used divergent thinking tasks, Dietrich and Kanso (2010) highlighted the importance of the prefrontal cortex (PFC) without pinpointing a specific sub-region. Insight problem-solving tasks usually require one right answer, which allows rating responses as correct or incorrect. In these tasks, the solution often comes to mind with insight (an “eureka” or “aha” moment). Combining words that are remotely semantically related can also evoke an “aha” experience. One popular example of such a combination task is the Remote Associates Task, which consists of finding a word that links three stimulus words, for example, finding the word “cheese” for “rat,” “blue,” and “cottage” (Mednick et al., 1964b). Functional neuroimaging that uses these tasks has focused on this “aha” aspect rather than on the combinatorial or associative processes that lead to a solution. Dietrich and Kanso (2010) reviewed 11 electrophysiological and nine functional imaging studies on insight, including the Remote Associates Task, and highlighted the frequent involvement of the superior temporal gyrus (STG) and anterior cingulate cortex (ACC). Finally, six studies that examined creativity in the artistic domains of music, dancing, and painting, using ecological tasks were examined in the same review. No region was identified as necessary and sufficient for artistic creativity. Both prefrontal activation and deactivation were reported, possibly suggesting the existence of distinct types of creativity. Overall, these results are in agreement with two recent reviews of neuroimaging and electrophysiological studies of creativity (Arden et al., 2010; Fink and Benedek, 2013) that also highlighted the PFC region without converging to specific prefrontal sub-regions. Studies that used other methods in creativity research, such as voxel-based morphometry, diffusion weighted imaging, or cerebral blood flow (CBF) (Bechtereva et al., 2004; Chavez-Eakle et al., 2007; Jung et al., 2010a,b; Takeuchi et al., 2010a,b) showed a link between creative performance and the lateral frontal and parieto-temporal regions and their connections.

Despite the diversity of tasks and cognitive approaches used to measure creativity, its link with PFC activity is expected. A central role for the PFC during creative behavior is in agreement with cognitive theories according to which several prefrontal functions (such as flexibility, fluency, planning, or working memory) are key cognitive processes of creativity (Carlsson et al., 2000; Zeki, 2001; Dietrich, 2004; Mendez, 2004; Bogousslavsky, 2005; Changeux, 2005; Ward, 2007). However, the precise prefrontal sub-regions involved and their specific roles remain to be clarified. The brain location of functional imaging results was examined qualitatively in two previous reviews (Arden et al., 2010; Dietrich and Kanso, 2010), but not statistically. Consequently, the questions of whether creative thinking is statistically associated with particular sub-regions, and whether different aspects of creativity, measured by different tasks, can be related to distinct sub-regions, remain to be tested.

The aim of this meta-analysis was to identify both shared and unique neural correlates of creative thinking by performing a statistical comparison between multiple studies. We explored brain regions that are most consistently associated with creativity tasks in published functional imaging studies. The results are discussed in light of the data drawn from other methods, including patient studies. The observation of shared regions, despite the diversity of tasks and criteria used to measure creativity, would suggest the existence of a core network for creativity. In addition, in order to determine whether there are process-specific regions, experiments were categorized according to tasks (combination or unusual generation tasks) and stimuli (verbal or non-verbal). The latter distinction aimed at comparing the correlates of creativity in two distinct classical domains of cognition (verbal or non-verbal). The former distinction was based on two separate and influential cognitive theories of creativity mentioned above. The first theory emphasizes the importance of combinatorial processes in creative thinking (new combination of remote associates) and was operationalized in the Remote Associates Task by Mednick (Mednick, 1962; Mednick et al., 1964a,b; Ward and Kolomyts, 2010). Combination tasks involve associating separate and remote elements of information to form a new idea. The second theory, derived from Guilford's work (Guilford, 1950; Runco, 2010), focuses on the level of fluency, flexibility, and originality of generated ideas, and has mainly been operationalized using divergent thinking tasks, such as the Alternate Uses Task. Tasks in the unusual generation category thus consist of producing original or unusual responses to a given stimulus or situation.

## Methods

### Selection of the studies

Studies were all peer-reviewed and published in English before June 2012. The PubMed and Scopus Medline databases were searched using the following keywords in text and/or abstract/title and Boolean operators: “creativity, creative thinking, creative process, unusualness, hypothesis generation, idea generation, aha, eureka, novel ideas, original ideas, originality, insight problem-solving, insight solution, artistic” AND “brain imaging, cerebral imaging, MRI, fMRI, PET, neural correlates, cerebral correlates, brain activation, functional magnetic resonance.” In order to ensure that inclusion criteria was as unbiased as possible, we did not systematically search for studies on domains that may be relevant to creativity (such as imagination, metaphors, music improvisation or expression, mental imagery, counterfactual thinking), or studies that explore various processes presumably involved in creativity (such as cognitive flexibility, inhibition of prepotent responses, working memory, planning, and so on), but such studies were included if the authors related explicitly to creativity in their work.

In addition, in order to be included in the meta-analysis, studies had to meet the following inclusion criteria: (1) using functional imaging in healthy adults, (2) reporting whole-brain results of signal changes in stereotactic space in 3D coordinates (*x, y, z*) in the Montreal Neurological Institute space (MNI; Evans et al., 1993) or the Talairach space (Talairach and Tournoux, 1988), and (3) reporting the peak coordinates in these spaces. We reviewed activation contrasts between tasks performed during the scanning of one or several groups of participants. In each study, only independent contrasts were included. If several contrasts in the same study were dependent, only results from the contrast reporting the most significant maxima were included. Between-group comparisons based on level of expertise were not included because their interpretation is difficult in terms of neurocognitive processes.

We analyzed a total of 443 activation foci reported in 44 independent contrasts from 34 experiments carried out in 622 healthy participants, from studies listed in Table 1.

**Table 1:** **List of the included studies with task description and categorization**.

**Authors**	**Year**	***N* subjects**	**Task description**	**Domain of material**	**Task type**
Abraham et al. (contrast 1)	2012b	19	Alternate Uses task (for objects) vs. fluency for locations	Verbal	Unusual generation
Abraham et al. (contrast 2)	2012b	Same as above	Alternate Uses task (for objects) and fluency for locations vs. 2-back and 1-back	Verbal	Unusual generation
Asari et al.	2008	68	Rorschach-like test: comparison of “unique” vs. “frequent” responses	Non-verbal^§^	Unusual generation
Aziz-Zadeh et al.	2009	10	Anagram solving task in experts: comparison of Aha vs. non Aha responses	Verbal	None
Aziz-Zadeh et al.	2013	13	Assembling three distinct shapes to form a new one: comparison of creative vs. basic conditions	Non-verbal^§^	Combination
Bechtereva et al.	2004	16	Creation of stories from a set of 16 remote words vs. memorize words	Verbal	None
Bechtereva et al.	2004	9	Produce associative verbal links between words vs. words reading	Verbal	Combination
Bengtsson et al.	2007	11	Music improvisation vs. play from memory in professional pianists	Non-verbal	None
Berkowitz and Ansari (contrast 1)	2008	13	Music improvisation in classical pianists: melodic improvisation vs. patterns	Non-verbal^♪^	Unusual generation
Berkowitz and Ansari (contrast 2)	2008	Same as above	Music improvisation in classical pianists: rhythmic improvisation vs. metronome	Non-verbal^♪^	Unusual generation
Cardillo et al.	2012	20	Comprehension of metaphors: novel vs. familiar metaphors	Verbal	Combination
Chrysikou and Thompson-Schill	2011	24	Alternate Uses task: generation of unusual vs. usual uses for objects	Verbal	Unusual generation
de Manzano and Ullén	2012	18	Music improvisation in classical pianists vs. music reading	Non-verbal^♪^	None
Ellamil et al.	2012	15	Design of book cover illustrations: ideas generation vs. evaluation phases	Non-verbal^§^	None
Fink et al. (contrast 1)	2010	31	Alternate Uses task: alternative uses vs. object characteristics	Verbal	Unusual generation
Fink et al. (contrast 2)	2010	Same as above	Alternate Uses task: incubation vs. no incubation phase	Verbal	Unusual generation
Fink et al. (contrast 3)	2010	Same as above	Alternate Uses task: stimulation with others ideas vs. no stimulation	Verbal	Unusual generation
Fink et al. (contrast 1)	2009	21	Alternate Uses task vs. fixation	Verbal	Unusual generation
Fink et al. (contrast 2)	2009	Same as above	Name invention vs. fixation	Verbal	Unusual generation
Geake and Hansen	2005	12	Fluid letter string analogy tasks: effect of analogical depth	Verbal	Combination
Goel and Vartanian (contrast 1)	2005	13	Match problems task vs. baseline	Non-verbal^§^	None
Goel and Vartanian (contrast 2)	2005	Same as above	Match problems task: successful vs. unsuccessful	Non-verbal^§^	None
Goel and Vartanian (contrast 3)	2005	Same as above	Match problems task: positive correlation with the number of solutions	Non-verbal^§^	None
Green et al. (contrast 1)	2012	23	Analogy task: effect of semantic distance	Verbal	Combination
Green et al. (contrast 2)	2012	Same as above	Analogy task: generation vs. rest	Verbal	Combination
Howard-Jones et al. (contrast 1)	2005	8	Story generation from a set of three words: creative vs. uncreative condition	Verbal	None
Howard-Jones et al. (contrast 2)	2005	Same as above	Story generation from a set of three words: unrelated vs. related words in the set	Verbal	Combination
Huang et al.	2012	26	Imagination of pictures based on given clues: creative (imagine novel and interesting pictures) vs. uncreative (figure out a common pattern not necessarily unique)	Non-verbal^§^	Unusual generation
Jung-Beeman et al.	2004	18	Compound remote-associates problem: Aha vs. no Aha	Verbal	Combination
Kounios et al.	2006	25	Compound remote-associates problem:—aha vs. no aha during preparation phase (before cues display)	Verbal	None
Kowatari et al. (study 1)	2009	20	Design of a new tool (a pen) by experts	Non-verbal^§^	Unusual generation
Kowatari et al. (study 2)	2009	20	Design of a new tool (a pen) by novices	Non-verbal^§^	Unusual generation
Kröger et al.	2012	19	Modified Alternate Uses Task (Conceptual expansion: judgment of word pairs according to unusualness and appropriateness)	Verbal	Combination
Limb and Braun	2008	6	Music improvisation vs. over-learned (jazz and scale) in expert pianists	Non-verbal^♪^	None
Luo et al.	2004	15	Solving ambiguous sentences with solution cues: aha vs. no aha	Verbal	Combination
Mashal et al.	2007	15	Metaphor: novel metaphors vs. unrelated words	Verbal	Combination
Qiu et al.	2010	16	Chinese logogriphs: Aha vs. no Aha problem solving	Verbal	Combination
Rutter et al.	2012	18	Conceptual expansion (judgment of sentences according to unusualness and appropriateness)	Verbal	Combination
Seger et al.	2000	7	Noun-verb generation task: unusual vs. first associate	Verbal	Unusual generation
Shah et al. (contrast 1)	2011	28	“Creative story writing”: writing a creative continuation for a text	Verbal	None
Shah et al. (contrast 2)	2011	Same as above	“Brainstorming”: thinking of a creative continuation for a text	Verbal	None
Siebörger et al.	2007	14	Graded coherence judgment task between sentences: distant vs. unrelated judgment	Verbal	Combination
Tian et al.	2011	16	Chinese logogriphs—preparation phase of successful vs. unsuccessful problem solving	Verbal	None
Vartanian and Goel	2005	15	Anagrams: unconstrained (no indices) vs. semantically constrained (given a semantic category)	Verbal	None

§ visual domain;

♪ music.

### Contrast categories (Table 1)

Each study was categorized in order to look for dissociations between networks associated with distinct creativity domains or operations. As it was difficult to group heterogeneous tasks in categories based on task used, they were classified into larger categories based on type of process involved (for example, combination of information vs. self-generation of unusual responses) and domain of information processing (verbal or not) used. First, we categorized each experiment according to the type of creativity processes: combination or free unusual generation tasks. Tasks that involved an explicit request to freely generate an unusual response were gathered in the “unusual generation” category, while those that required the combination of separate and remote elements were categorized as “combination.” Tasks that did not fall into one of these categories were not included, leaving 29 experiments in this sub-analysis.

The second classification was based on the verbal or non-verbal nature of the stimuli used. The non-verbal category included visual and musical domains. While these domains are different, they were gathered into the non-verbal category because the number of experiments in each domain separately was insufficient for statistical testing.

All contrasts were classified according to these categories by two double-blind authors (GGY, EV). The few disagreements that occurred were all solved by discussion between the co-authors.

### ALE methods

#### General principles

We performed a meta-analysis of functional neuroimaging data on creativity using Activation Likelihood Estimation (GingerALE) software (http://brainmap.org/ale/cli.html; Laird et al., 2005; Eickhoff et al., 2009, 2012; Turkeltaub et al., 2012). ALE is a coordinate-based meta-analysis method that uses published activation peaks reported in functional imaging studies in a normalized coordinate referential. ALE delineates the regions in the brain where convergence across all included studies is higher than would be expected by chance (null distribution of randomly generated activation likelihoods) (Eickhoff et al., 2009). In other words, ALE evaluates the “inter-experiment” reliability of the involvement of brain regions in given processes—in this case in creativity tasks.

#### Global analysis

The ALE analyses were conducted using the GingerALE software v2.2 (www.brainmap.org; Eickhoff et al., 2009, 2012; Turkeltaub et al., 2012). Coordinates collected from studies that were reported in Talairach space were converted into the MNI space using the tal2mni algorithm implemented in Matlab (http://imaging.mrc-cbu.cam.ac.uk/imaging/MniTalairach). In the first step, activation foci from each included study were modeled as Gaussian distributions and merged into a single 3D volume. To address the problem of the independence of observation within the same study, we used the modified ALE algorithm (Turkeltaub et al., 2012) and organized datasets according to subject groups. The algorithm also modeled spatial uncertainty (Eickhoff et al., 2009, 2012)—and thus probability distribution—of each focus, using an estimation of the inter-subject and inter-laboratory variability typically observed in neuroimaging experiments, rather than a pre-specified full-width half maximum (FWHM). Thus, the number of participants in a given study influenced the spatial extent of the Gaussian function used. GingerALE first modeled the probability of activation over all studies at each spatial point in the brain, returning localized “activation likelihood estimates” or ALE values. In a second step, ALE values were compared to a null distribution created from simulated datasets with randomly placed foci in order to identify significantly activated clusters. ALE maps were calculated using 10,000 permutations. We used a cluster correction for multiple comparisons (Eickhoff et al., 2012) with a false discovery rate (FDR) corrected threshold at *p* < 0.05 for cluster-formation and then a *p* < 0.05 for cluster thresholding. Only clusters with a size exceeding the cluster size recommended by ALE were reported. We used an extent-threshold because cluster-level inference may represent a compromise between uncorrected thresholding with additional arbitrary extent correction and voxel-level corrected inference. Moreover, cluster-level thresholding seems to provide a better balance between sensitivity and specificity than the highly conservative voxel-level family-wise error (FWE) correction (Eickhoff et al., 2012).

#### Focused sub-analyses

In order to analyse specific task categories, an ALE analysis was first performed separately for each task category (combination and unusual generation tasks, verbal and non-verbal). We proceeded as described for the global analysis (with a cluster thresholding), but entered only selected corresponding foci.

#### Task comparisons

Differences between task categories were tested by first performing an ALE analysis separately for each condition (thresholded at *p* < 0.05 uncorrected) and then computing the voxel-wise difference between the resulting ALE maps (Laird et al., 2005). The difference in ALE value between two ALE maps was computed at each voxel, and statistical significance was tested using permutations. An FDR correction at *p* < 0.05 was used with a minimum cluster size of 200 mm^3^ in order to address the problem of multiple comparisons.

#### ALE results

Anatomical labels of final cluster locations were provided by the Talairach Daemon (http://www.talairach.org/daemon.html) and available as a GingerALE output. Each ALE map was visualized using Mango (http://ric.uthscsa.edu/mango) and Anatomist (http://brainvisa.info/), and was overlaid on the anatomical Colin27 Template for visual inspection and representation purposes using Anatomist.

## Results

### All studies (see Table 2, Figure 1)

The global ALE map revealed a network consistently associated with various creativity tasks, including the bilateral inferior and left superior PFC (BA 44, 47, 46, 9, 10), the medial PFC (BA 6, 9), the bilateral ACC and insula, the left anterior (BA 37) and posterior lateral temporal gyri (BA 22, 37), the right fusiform gyrus, the left supramarginal gyrus (BA 40) and precuneus (BA 7), the bilateral occipital cortex, the bilateral anterior and posterior cerebellum, and the left thalamus.

**Table 2:** **Locations of clusters with significant ALE values for the global analysis**.

**Location**		**Left**					**Right**				
	**BA**	**Cluster number and size (mm3)**	**ALE**	***x***	***y***	***z***	**Cluster number and size (mm3)**	**ALE**	***x***	***y***	***z***
**FRONTAL LOBE**											
Inferior frontal G	9	1 (59080)	0.0258	−44	10	26					
Inferior frontal G	44	1 (59080)	0.0242	−48	18	8	3 (5424)	0.0157	54	14	10
Inferior frontal G	47	1 (59080)	0.0130	−40	36	−6	10 (1960)	0.0094	46	24	−8
Inferior frontal G	46	1 (59080)	0.0138	−44	46	0	10 (1960)	0.0090	50	38	4
Middle frontal G	8	1 (59080)	0.0201	−20	36	42					
Middle frontal G	6						11 (1688)	0.0177	26	−4	50
Middle frontal G	46	1 (59080)	0.0096	−48	36	14	10 (1960)	0.0079	48	46	8
Middle frontal G	9						3 (5424)	0.0183	48	18	28
Middle frontal G	46						3 (5424)	0.0172	50	22	24
Middle frontal G	10	1 (59080)	0.0095	−42	48	−12					
Middle frontal G	10	9 (2704)	0.0092	−30	52	20					
Medial frontal G	10	9 (2704)	0.0113	−8	62	10					
Medial frontal G	9	9 (2704)	0.0091	−18	50	10					
Superior frontal G	10	9 (2704)	0.0093	−18	52	18					
Superior frontal G	6						1 (59080)	0.0152	2	24	58
Precentral G	6	1 (59080)	0.0167	−36	2	44	3 (5424)	0.0155	48	8	32
Precentral G	4	1 (59080)	0.0115	−36	−18	56					
**CINGULATE G**											
Cingulate G	32	1 (59080)	0.0232	−8	28	30					
**INSULA**											
Insula	13	1 (59080)	0.0216	−44	16	−2	10 (1960)	0.0090	40	24	−6
**TEMPORAL LOBE**											
Superior temporal G	38	1 (59080)	0.0129	−50	14	−22					
Superior temporal G	22	2 (8224)	0.0204	−58	−42	12					
Fusiform G	37	2 (8224)	0.0183	−50	−50	−14	7 (3928)	0.0146	44	−52	−16
Middle temporal G	22	2 (8224)	0.0111	−54	−46	0					
Inferior temporal G	37	2 (8224)	0.0079	−56	−60	−6					
Fusiform G	18	6 (3944)	0.0154	−20	−94	−12	5 (3968)	0.0189	22	−94	−8
Middle temporal G	19	8 (3000)	0.0157	−50	−64	20					
Middle temporal G	39	8 (3000)	0.0077	−58	−66	30					
**PARIETAL LOBE**											
Inferior parietal lob.	40	1 (59080)	0.0150	−44	−40	44	12 (1568)	0.0165	42	−40	44
Supramarginal G	40	1 (59080)	0.0109	−56	−38	38					
Postcentral G	3	1 (59080)	0.0126	−36	−26	50					
Precuneus	7	4 (5416)	0.0155	−28	−66	44					
Supramarginal G	40	8 (3000)	0.0155	−48	−52	24					
**OCCIPITAL LOBE**											
Superior occipital G	19	4 (5416)	0.0163	−40	−80	36					
Inferior occipital G	18						5 (3968)	0.0139	32	−92	−4
Middle occipital G	18	6 (3944)	0.0129	−38	−88	−6	5 (3968)	0.0110	34	−88	2
**CEREBELLUM**											
Culmen		2 (8224)	0.0177	−30	−58	−24	7 (3928)	0.0092	36	−56	−24
Tuber		2 (8224)	0.0142	−46	−66	−24					
Declive		6 (3944)	0.0096	−24	−84	−18					
Posterior lobe							7 (3928)	0.0177	34	−68	−22
Cerebellar tonsil							7 (3928)	0.0084	38	−70	−36
**SUB-CORTICAL**											
Thalamus		13 (1240)	0.0110	−10	−18	12					

Columns number 3–7 represent data associated with the left hemisphere and 7–12 represent data associated with the right hemisphere. Abbreviations: BA, approximate Brodmann area; ALE, activation likelihood estimation; G, gyrus; Lob, lobule; x, y, z coordinates, peak voxel in the Montreal Neurologic Institute (MNI) space.

**Figure 1:**
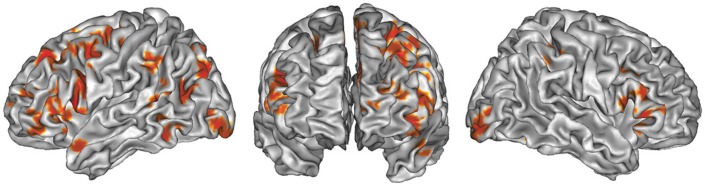
**ALE map of all foci**, thresholded at a whole-brain cluster-level corrected *p* < 0.05. Activations are displayed on the surface rendering of the Colin27 template (Holmes et al., 1998) in the MNI space.

### Combination vs. unusual generation tasks (Tables 3, 4, 5 and Figures 2A,B)

**Figure 2:**
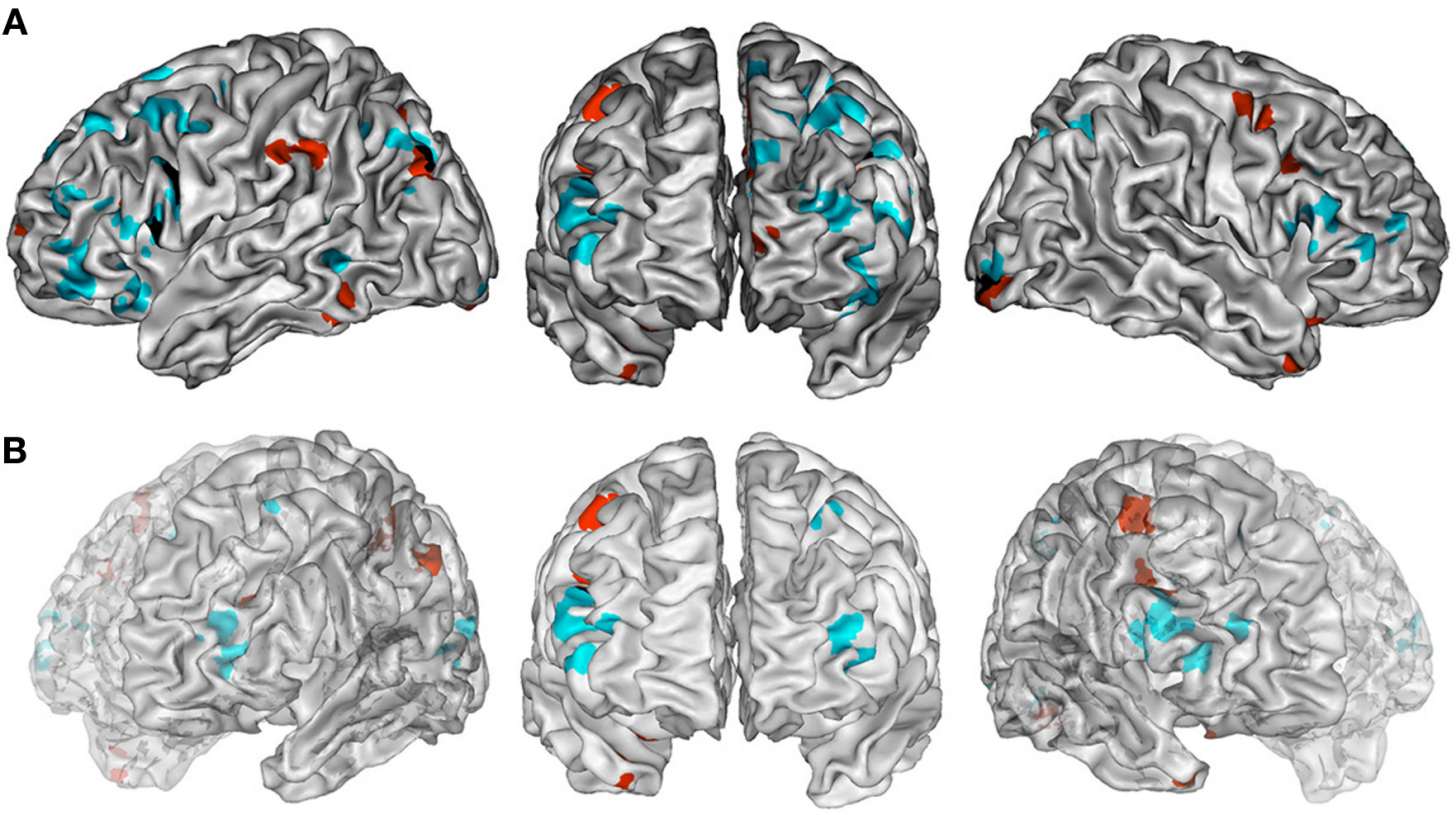
**(A)** ALE maps of combination task foci (in cyan) and generation task foci (in red). Overlaps between maps are shown in black. All maps were thresholded at a whole-brain cluster-level corrected *p* < 0.05. **(B)** ALE maps resulting from contrast studies of combination vs. generation tasks foci (in cyan) and generation vs. combination task foci (in red). These contrast maps were thresholded at a whole-brain FDR corrected *p* < 0.05. In **(A,B)**, ALE maps are displayed on a surface rendering of the Colin27 template (Holmes et al., 1998) in the MNI space.

The ALE map that resulted from grouping combination tasks (Figure 2A, Table 3) revealed a bilateral and predominantly left network involving the lateral PFC (BA 45, 47, 46), including its rostrolateral part (BA 10), the left precentral region (BA 6), the left ACC (BA 24/32), the bilateral insula, the posterior temporal gyri (BA 22, 39), the left inferior parietal lobule (BA 40), the right superior parietal lobule (BA 7) and bilateral precuneus (BA 7), the fusiform and lingual gyri, and the cerebellum (lobule VI).

**Table 3:** **Locations of clusters with significant ALE values for combination tasks**.

**Location**		**Left**					**Right**				
	**BA**	**Cluster number and size (mm3)**	**ALE**	***x***	***y***	***z***	**Cluster number and size (mm3)**	**ALE**	***x***	***y***	***z***
**FRONTAL LOBE**											
Middle frontal G	10	1 (15416)	0.0095	−42	48	−12	10 (1184)	0.0077	40	50	14
Middle/Superior frontal G	10	6 (1776)	0.0090	−30	52	20					
Middle frontal G	46	1 (15416)	0.0083	−36	36	4	4 (3504)	0.0103	54	24	18
Middle frontal G	9	1 (15416)	0.0083	−52	14	36	4 (3504)	0.0087	50	22	26
Inferior frontal G	9	1 (15416)	0.0161	−44	8	30	10 (1184)	0.0086	50	38	4
Inferior frontal G	45	1 (15416)	0.0130	−50	24	12					
Inferior frontal G	47	1 (15416)	0.0086	−36	30	−8					
Inferior frontal G	44						4 (3504)	0.0081	54	16	12
Superior frontal G	8	12 (816)	0.0087	−18	38	46					
Superior frontal G	6	7 (1544)	0.0102	−2	18	64					
Medial frontal G	6	2 (5608)	0.0193	−6	32	32					
Medial frontal G	6	5 (3472)	0.0141	−20	14	52					
Medial frontal G	8	15 (656)	0.0084	−6	58	40					
Precentral G	6/44	1 (15416)	0.0079	−42	2	44	4 (3504)	0.0082	60	16	4
Precentral G	6	16 (624)	0.0075	−32	−2	58					
**CINGULATE CORTEX**											
Cingulate G	32	2 (5608)	0.0145	0	14	40	2 (5608)	0.0087	6	12	42
Anterior cingulate	24	2 (5608)	0.0084	−8	26	22					
**INSULA**											
Insula	13	1 (15416)	0.0130	−38	18	8	4 (3504)	0.0077	38	18	10
**TEMPORAL LOBE**											
Lingual G	18						13 (752)	0.0078	20	−96	−10
Middle temporal G	39	17 (520)	0.0075	−50	−74	26					
Middle temporal G	22	8 (1488)	0.0109	−54	−46	0					
Superior temporal G	22	8 (1488)	0.0086	−56	−42	10					
**PARIETAL LOBE**											
Inferior parietal lobule	40	3 (3864)	0.0079	−40	−52	44					
Precuneus	19	3 (3864)	0.0080	−40	−78	42	9 (1264)	0.0075	30	−72	44
Precuneus	39	3 (3864)	0.0124	−32	−64	42					
Superior parietal lobule	7						9 (1264)	0.0126	30	−62	46
**OCCIPITAL LOBE**											
Superior occipital G	39	3 (3864)	0.0101	−30	−74	34					
Inferior occipital G	18						11 (880)	0.0123	34	−94	−4
Fusiform G	18	14 (704)	0.0082	−20	−98	−14					
**CEREBELLUM**											
Declive		14 (704)	0.0075	−16	−92	−18					

Columns number 3–7 represent data associated with the left hemisphere and 7–12 represent data associated with the right hemisphere. Abbreviations: BA, approximate Brodmann area; ALE, activation likelihood estimation; G, gyrus; x, y, z coordinates, peak voxel in the Montreal Neurologic Institute (MNI) space.

**Table 4:** **Locations of clusters with significant ALE values for unusual generation tasks**.

**Location**		**Left**					**Right**				
	**BA**	**Cluster number and size (mm3)**	**ALE**	***x***	***y***	***z***	**Cluster number and size (mm3)**	**ALE**	***x***	***y***	***z***
**FRONTAL LOBE**											
Inferior frontal G	9	1 (6176)	0.0138	−46	12	26					
Inferior frontal G	44	1 (6176)	0.0078	−46	20	8					
Inferior frontal G	47						16 (1000)	0.0103	32	18	−24
Middle frontal G	46	1 (6176)	0.0096	−42	22	20					
Medial frontal G	6	7 (1688)	0.0160	−4	8	52					
Medial frontal G	10	12 (1096)	0.0103	−8	62	10					
Precentral G	6	1 (6176)	0.0140	−38	2	34	14 (1056)	0.0124	44	−2	54
Precentral G	9/6						11 (1136)	0.0111	44	10	32
**CINGULATE CORTEX**											
Cingulate G	24	5 (2088)	0.0096	−2	18	32					
Cingulate G	24	19 (656)	0.0069	−16	0	48	18 (832)	0.0080	0	−6	36
Cingulate G	32	5 (2088)	0.0094	0	20	36					
Cingulate G	31						17 (928)	0.0089	6	−36	28
Cingulate G	23						17 (928)	0.0086	10	−28	30
**TEMPORAL LOBE**											
Fusiform G	37	8 (1512)	0.0133	−50	−50	−16	3 (2576)	0.0089	42	−52	−16
Fusiform G	18						9 (1416)	0.0094	22	−94	−10
Superior temporal G	38						16 (1000)	0.0076	36	16	−34
**PARIETAL LOBE**											
Inferior parietal lob.	40	4 (2400)	0.0114	−42	−36	44					
Supramarginal G	40	15 (1016)	0.0092	−60	−28	36					
Precuneus	7	10 (1312)	0.0117	−22	−66	46					
**OCCIPITAL LOBE**											
Superior occipital G	19	13 (1080)	0.0132	−40	−80	34					
Inferior occipital G	18						9 (1416)	0.0088	26	−92	−8
**CEREBELLUM**											
Culmen		2 (3080)	0.0115	−24	−62	−24	3 (2576)	0.0089	36	−56	−26
Culmen		6 (1712)	0.0084	−20	−32	−16					
Declive							3 (2576)	0.0120	34	−68	−24
**SUB-CORTICAL**											
Thalamus		6 (1712)	0.0132	−16	−30	−4					

Columns number 3–7 represent data associated with the left hemisphere and 7–12 represent data associated with the right hemisphere. Abbreviations: BA, approximate Brodmann area; ALE, activation likelihood estimation; G, gyrus; x, y, z coordinates, peak voxel in the Montreal Neurologic Institute (MNI) space.

**Table 5:** **Locations of clusters with significant ALE values for the contrast of combination vs. generation tasks and the reverse contrast**.

**Location**		**Left**					**Right**				
	**BA**	**Cluster number and size (mm3)**	**ALE**	***x***	***y***	***z***	**Cluster number and size (mm3)**	**ALE**	***x***	***y***	***z***
**COMBINATION vs. GENERATION TASKS**											
**FRONTAL LOBE**											
Inferior frontal G	45						1 (5896)	2.1248	53	22.1	8.4
Inferior frontal G	45						2 (1616)	2.1248	51.7	36.5	4.3
Middle frontal G	10	3 (944)	2.6693	−40	50.5	5.5					
Middle frontal G	10	5 (784)	3.0902	−36	52	8					
Inferior frontal G	10	3 (944)	1.7224	−38	46	−6					
Middle frontal G	6	8 (216)	1.8564	−32	18	56					
**INSULA**											
Insula	13						1 (5896)	2.1272	34	22	8
**TEMPORAL LOBE**											
Middle temporal G	21	4 (920)	1.7841	−56.9	−45.3	6					
Middle temporal G	37	6 (672)	2.1444	−56.7	−60.9	12					
**PARIETAL LOBE**											
Angular G	39	7 (448)	2.2383	−44	−64	38					
Inferior parietal lob	39	7 (448)	2.2173	−47	−66	44					
Precuneus	39	7 (448)	2.0122	−36	−70	40					
**GENERATION vs. COMBINATION TASKS**											
**FRONTAL LOBE**											
Middle frontal G	9	7 (568)	2.2571	−30.7	27.3	24					
**CINGULATE CORTEX**											
Posterior cingulate	29						5 (712)	2.0047	23	−38.1	18.6
**PARIETAL LOBE**											
Supramarginal G	40	3 (2448)	1.7324	−46.2	−40.7	40. 9					
Inferior parietal lobule	40	3 (2448)	2.4677	−43.7	−29.7	45.9					
Inferior parietal lobule	40	4 (896)	2.4677	−56	−28	40					
**CEREBELLUM**											
Culmen		1 (4960)	3.0618	−31.3	−55	−26.7	2 (3224)	2.9677	38.5	−52.5	−18.8
Culmen							6 (640)	2.7266	25	−48	−19
Tuber							8 (488)	1.9566	55	−55	−28
Anterior lobe		1 (4960)	2.9478	−30	−48.6	−19.8	2 (3224)	1.9431	37	−59	−32.7
Declive							2 (3224)	2.4522	38	−68	−18
**SUB-CORTICAL**											
Thalamus		9 (240)	1.9991	−19	−28	2	5 (712)	1.7542	18	−36	14

Columns number 3–7 represent data associated with the left hemisphere and 7–12 represent data associated with the right hemisphere. Abbreviations: BA, approximate Brodmann area; ALE, activation likelihood estimation; x, y, z coordinates, peak voxel in the Montreal Neurologic Institute (MNI) space.

The ALE map that resulted from grouping unusual generation tasks (Figure 2A, Table 4) revealed a network that included the left inferior and middle frontal gyrus (BA 9, 44, 46), the left rostromedial PFC (BA 10), the bilateral precentral gyrus (BA 6), the left anterior and right posterior cingulate cortex, the bilateral fusiform gyrus (BA 37), the right temporal pole (BA 38), the left inferior parietal lobule (BA 40) and precuneus (BA 7), bilateral cerebellar lobules IV and V, the occipital cortex, and the left thalamus.

Combination and generation tasks overlapped in several focused regions, including the inferior frontal junction (IFJ), the inferior frontal gyrus (IFG), the posterior middle frontal gyrus, the parieto-occipital cortex, and the medial wall (Figure 2A).

When comparing these two task categories statistically (Figure 2B, Table 5), ALE showed regions more consistently associated with combination than generation tasks. These regions were located in the left rostrolateral PFC (BA 10) and the left inferior and middle frontal gyri (BA 45, 46), in the right IFG (BA 45, 46) and insula, in the left posterior middle temporal gyrus (BA 21/37), and in the left posterior parietal region.

Conversely, ALE showed regions that were more strongly associated with generation than combination tasks within the bilateral cerebellum (Lobules IV, V, VI, and VIIb), the bilateral thalamus, the left inferior parietal lobule (BA 40), the right posterior cingulate (BA 29), and the left middle frontal gyrus (BA 9).

### Verbal vs. non-verbal tasks (Tables 6, 7, 8 and Figures 3A,B)

**Figure 3:**
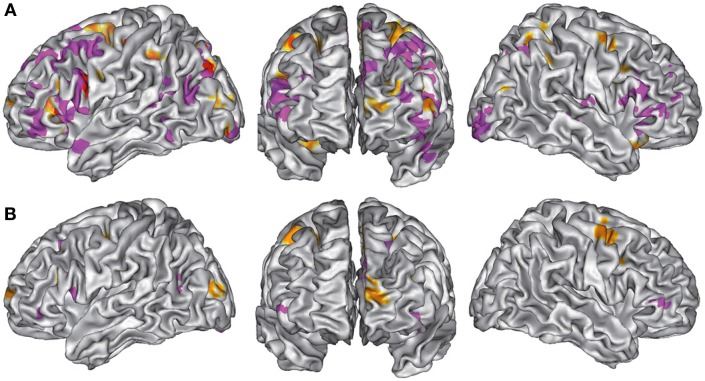
**(A).** ALE maps of verbal task foci (in purple) and non-verbal task foci (in orange). Overlaps between maps are shown in red. All maps were thresholded at a whole-brain cluster-level corrected *p* < 0.05. **(B)**. ALE maps resulting from contrast studies of verbal vs. non-verbal task foci (in purple) and non-verbal vs. verbal task foci (in orange). These contrast maps were thresholded at a whole-brain FDR corrected *p* < 0.05. In **(A,B)**, ALE maps are displayed on a surface rendering of the Colin27 template (Holmes et al., 1998) in the MNI space.

For verbal tasks only (Figure 3A, Table 6), significant activation was found bilaterally with a left dominance within lateral prefrontal regions, including the IFG (BA 44, 47), the middle frontal gyrus (BA 8, 9, 46), and extending into the rostral PFC (medial and lateral BA 10), and the superior frontal gyrus (BA 6, 8). Additional regions were observed in the left anterior temporal fusiform gyrus and in the posterior part of the lateral temporal region extending into the inferior parietal lobule (BA 39/40), in the middle temporal gyrus caudally, in the left fusiform gyrus, and the anterior STG. The bilateral insula, superior parietal lobule, cerebellum, and subcortical structures were also involved.

**Table 6:** **Locations of clusters with significant ALE values for verbal tasks**.

**Location**		**Left**					**Right**				
	**BA**	**Cluster number and size (mm3)**	**ALE**	***x***	***y***	***z***	**Cluster number and size (mm3)**	**ALE**	***x***	***y***	***z***
**FRONTAL LOBE**											
Inferior frontal G	44	1 (37792)	0.0240	−48	18	8	4 (3832)	0.0097	62	12	12
Inferior frontal G	47	1 (37792)	0.0128	−40	36	−6	7 (2552)	0.0091	46	24	−8
Inferior frontal G	9	1 (37792)	0.0176	−44	8	30					
Inferior frontal G	46	1 (37792)	0.0137	−44	46	0	7 (2552)	0.0090	50	38	4
Middle frontal G	10	1 (37792)	0.0095	−42	48	−12	7 (2552)	0.0078	40	50	14
Middle frontal G	10	20 (816)	0.0089	−34	50	14					
Middle frontal G	46	1 (37792)	0.0081	−46	32	16	4 (3832)	0.0170	50	22	24
Middle frontal G	9	1 (37792)	0.0087	−52	14	36					
Middle frontal G	8	1 (37792)	0.0124	−32	24	44					
Middle frontal G	8	9 (1672)	0.0197	−20	36	42	21 (808)	0.0126	32	44	34
Superior frontal G	6						1 (37792)	0.0127	2	24	58
Superior frontal G	8	19 (848)	0.0096	−10	52	36					
Medial frontal G	6						1 (37792)	0.0223	−6	22	42
Precentral G	4	8 (3276)	0.0088	−36	−18	56					
**INSULA**											
Insula	13	1 (37792)	0.0204	−42	20	4	7 (2552)	0.0089	40	24	−6
**CINGULATE G**											
Cingulate G	32	1 (37792)	0.0231	−8	28	30					
Cingulate G	24	1 (37792)	0.0089	−2	18	30					
Cingulate G	31	23 (744)	0.0121	−4	−46	32					
**TEMPORAL LOBE**											
Superior temporal G	38	1 (37792)	0.0129	−50	14	−22					
Superior temporal G	22	12 (1432)	0.0160	−56	−40	10	11 (1520)	0.0094	50	−26	0
Middle temporal G	19	5 (3248)	0.0156	−50	−64	20					
Middle temporal G	39	5 (3248)	0.0076	−58	−66	30					
Middle temporal G	22	12 (1432)	0.0092	−48	−40	6					
Fusiform G	18	6 (2568)	0.0147	−20	−96	−12	2 (4216)	0.0189	22	−94	−8
Fusiform G	37	1248	0.0134	−50	−50	−16					
**PARIETAL LOBE**											
Precuneus	7	3 (3920)	0.0080	−18	−76	48					
Precuneus	19	3 (3920)	0.0152	−30	−64	44	18 (864)	0.0075	30	−72	44
Supramarginal G	40	5 (3248)	0.0155	−48	−52	24					
Inferior parietal lobule	40	8 (3276)	0.0118	−40	−32	46					
Postcentral G	3	8 (3276)	0.0125	−36	−26	50					
Superior parietal lobule	7						18 (864)	0.0126	30	−62	46
**OCCIPITAL LOBE**											
Inferior occipital G	18	6 (2568)	0.0079	−28	−94	−12	2 (4216)	0.0139	32	−92	−4
Superior occipital G	19	3 (3920)	0.0111	−36	−78	34					
**SUB-CORTICAL**											
Thalamus		10 (1600)	0.0110	−10	−18	12					
Lentiform nucleus							11 (1520)	0.0100	34	−16	8
Putamen		14 (1296)	0.0105	−32	−12	2					
Lateral globus pallidus		14 (1296)	0.0087	−24	−6	−10					
Medial globus pallidus		14 (1296)	0.0082	−16	−2	−10					
**CEREBELLUM**											
Cerebellar tonsil							13 (1376)	0.0083	38	−70	−36
Declive		6 (2568)	0.0093	−24	−84	−18	13 (1376)	0.0141	36	−66	−24
Declive							22 (776)	0.0121	8	−74	−22
Tuber		16 (1208)	0.0113	−46	−66	−28					
Inferior Semi−Lunar							17 (1048)	0.0104	30	−76	−42
Pyramis							17 (1048)	0.0084	30	−86	−34
Culmen		25 (672)	0.0098	−28	−58	−24	24 (712)	0.0111	20	−48	−18

Columns number 3–7 represent data associated with the left hemisphere and 7–12 represent data associated with the right hemisphere. Abbreviations: BA, approximate Brodmann area; ALE, activation likelihood estimation; G, gyrus; x, y, z coordinates, peak voxel in the Montreal Neurologic Institute (MNI) space.

**Table 7:** **Locations of clusters with significant ALE values for non-verbal tasks**.

**Location**		**Left**					**Right**				
	**BA**	**Cluster number and size (mm3)**	**ALE**	***x***	***y***	***z***	**Cluster number and size (mm3)**	**ALE**	***x***	***y***	***z***
**FRONTAL LOBE**											
Superior frontal G	6	1 (12432)	0.0068	−26	6	66					
Superior frontal G	6	2 (4184)	0.0075	−2	20	56					
Middle frontal G	46	1 (12432)	0.0112	−42	24	22					
Middle frontal G	46	5 (1984)	0.0082	−50	38	12					
Middle frontal G	9	15 (1136)	0.0083	−26	42	26					
Middle frontal G	6	1 (12432)	0.0068	−34	2	46	3 (2432)	0.0108	24	−6	50
Inferior frontal G	9	1 (12432)	0.0095	−44	10	26					
Inferior frontal G	47	5 (1984)	0.0075	−46	26	−6	6 (1688)	0.0116	32	18	−24
Medial frontal G	10	9 (1536)	0.0112	−8	62	10					
Medial frontal G	6	2 (4184)	0.0182	−2	6	54					
Precentral G	4/6	1 (12432)	0.0137	−38	2	34	14 (1192)	0.0124	44	−2	54
Precentral G	9						7 (1688)	0.0122	46	10	32
**CINGULATE CORTEX**											
Cingulate G	24	1 (12432)	0.0072	−18	0	48					
**INSULA**											
Insula	13	5 (1984)	0.0082	−44	14	−2					
**PARIETAL LOBE**											
Supramarginal G	40	4 (2152)	0.0084	−38	−44	42					
Inferior parietal lobule	40	4 (2152)	0.0090	−48	−36	44	11 (1328)	0.0086	40	−42	44
Superior parietal lobule	7						17 (992)	0.0082	32	−58	56
Precuneus	7	22 (760)	0.0076	−20	−64	48					
**OCCIPITAL LOBE**											
Superior occipital G	19	16 (1048)	0.0103	−42	−80	36					
Middle occipital G	19	10 (1536)	0.0068	−36	−82	14	21 (784)	0.0051	36	−76	18
Middle occipital G	18	10 (1416)	0.0086	−28	−84	10					
Inferior occipital G	18	20 (832)	0.0073	−28	−90	−8					
**TEMPORAL LOBE**											
Fusiform G	37						18 (928)	0.0067	44	−52	−18
**CEREBELLUM**											
Pyramis							8 (1600)	0.0091	28	−68	−32
Declive							8 (1600)	0.0083	32	−70	−22
Culmen		12 (1224)	0.0087	−30	−60	−24	18 (928)	0.0080	36	−54	−24
**SUBCORTICAL**											
Thalamus		13 (1192)	0.0131	−16	−30	−4	19 (904)	0.0084	24	−28	2

Columns number 3–7 represent data associated with the left hemisphere and 7–12 represent data associated with the right hemisphere. Abbreviations: BA, approximate Brodmann area; ALE, activation likelihood estimation; G, gyrus; x, y, z coordinates, peak voxel in the Montreal Neurologic Institute (MNI) space.

**Table 8:** **Locations of clusters with significant ALE values for the contrast of verbal vs. non−verbal tasks and the reverse contrast**.

**Location**		**Left**					**Right**				
	**BA**	**Cluster number and size (mm3)**	**ALE**	***x***	***y***	***z***	**Cluster number and size (mm3)**	**ALE**	***x***	***y***	***z***
**VERBAL vs. NON VERBAL TASKS**											
**FRONTAL LOBE**											
Medial frontal G	9	1 (3464)	3.1214	−7.3	38	31.3					
Superior frontal G	8	1 (3464)	2.4624	−16	24	44					
Inferior frontal G	44	2 (1328)	2.3656	−46	20	8					
Inferior frontal G	47	2 (1328)	1.9936	−40	24	6					
Middle/Inferior frontal	46	4 (568)	1.8080	−40	46	0	5 (528)	2.0600	52	38	2
Middle/inferior frontal	47	4 (568)	1.9634	−40	38	−6					
Middle frontal G	10						5 (528)	1.9515	38	44	2
Medial frontal G	8	8 (264)	2.7065	−8	44	36					
**CINGULATE CORTEX**											
Cingulate G	32/6	1 (3464)	2.9677	−14	28	32					
Anterior cingulate	24	1 (3464)	2.4838	−10	26	18					
**TEMPORAL LOBE**											
Lingual G	18						3 (1056)	1.9157	17.3	−87.7	−7
Middle temporal G	39	6 (496)	1.8277	−54	−62	12					
Superior temporal G	22	6 (496)	1.7542	−52	−54	22					
Superior temporal G	39	6 (496)	1.7147	−50	−56	26					
**CEREBELLUM**											
Declive							3 (1056)	1.6757	21.5	−91.8	−16
**SUB-CORTICAL**											
Thalamus		7 (472)	1.9617	−6	−16.7	16.7					
Lentiform nucleus		9 (248)	1.6986	−9	−2	−3					
**NON VERBAL vs. VERBAL TASKS**											
**FRONTAL LOBE**											
Middle frontal G	6	4 (1848)	3.0902	−28.5	−11	47	1 (3488)	3.2905	39.3	2.7	56
Middle frontal G	9	6 (992)	2.3867	−36	22	26					
Middle frontal G	6	7 (856)	1.9349	28.5	−9.7	47.2	8 (584)	1.8055	42	8	38
Medial frontal G	6						2 (2272)	2.6875	2	2	58
Medial frontal G	10	3 (2040)	2.0476	−16	62	6					
Medial frontal G	9						3 (2040)	2.0992	2	63	16
Superior frontal G	10	3 (2040)	2.4324	−7.7	65	16.9					
Precentral G	6	8 (584)	2.0578	45	8.3	26.3	1 (3488)	1.8808	50	0	52
**OCCIPITAL LOBE**											
Middle occipital G	18	5 (1688)	1.9317	−25	−84	14					
Middle occipital G	19	5 (1688)	2.1701	−38	−86	16					

Columns number 3–7 represent data associated with the left hemisphere and 7–12 represent data associated with the right hemisphere. Abbreviations: BA, approximate Brodmann area; ALE, activation likelihood estimation; G, gyrus; x, y, z coordinates, peak voxel in the Montreal Neurologic Institute (MNI) space.

For non-verbal tasks only (Figure 3A, Table 7), significant activation was found bilaterally, but predominantly in the left inferior (BA 47) and superior (BA 9, 46) parts of the lateral PFC, left rostromedial PFC (BA10), right and left precentral and medial BA 6, left ACC and insula, right and left occipital cortex, inferior (BA 40) and right superior (BA 7) parietal lobules, right fusiform gyrus (BA 37), and cerebellum (lobules IV, V, VI, VIII).

Verbal and non-verbal tasks overlapped in several focused left regions, including the IFJ, the posterior IFG, the parieto-occipital cortex, the posterior middle frontal gyrus, the medial wall, and the cerebellum (Figure 3A).

When comparing these two task domains statistically (Figure 3B, Table 8), ALE revealed some regions to be more consistently associated with verbal tasks: mainly the left and right lateral PFC (BA 8, 9, 44, 46, 47, 10). Additional regions in the left ACC, the left posterior STG (BA 22/37), the right lingual gyrus, and the left thalamus were also observed.

The reverse contrast showed a few regions more associated with non-verbal than verbal tasks, within the right and left premotor regions (medial and lateral BA 6), the left middle frontal gyrus (BA 9), and the left occipital cortex.

## Discussion

### General features of the shared creativity network

To our knowledge, the present study is the first quantitative meta-analysis to focus on creativity tasks. It reveals, despite the variety of tasks employed (Table 1), a statistical convergence across experiments in a set of brain regions (Figure 1): the caudal part of the lateral PFC, both ventrally and dorsally, the medial and lateral portion of the left rostral PFC, the inferior parietal lobule, and the lateral temporal gyrus. In this set of brain regions, the PFC is of central importance. This finding is in agreement with previous reviews (Fink et al., 2007; Dietrich and Kanso, 2010) as well as with the small number of lesion studies that has examined the cerebral bases of creativity in neurological patients (Miller and Tippett, 1996; Reverberi et al., 2005; de Souza et al., 2010; Shamay-Tsoory et al., 2011; Abraham et al., 2012a). In particular, Shamay-Tsoory et al. (2011) and Abraham et al. (2012a) demonstrated that damage to the rostral PFC impaired performance on divergent creativity tests such as the Alternate Uses test and the Torrance Test of Creative Thinking (TTCT, Torrance, 1979). The present meta-analysis similarly pointed to the rostral PFC (BA 10) as an important region for creativity tasks.

This set of brain regions shared by functional imaging studies is also consistent with those observed using other methods, in both the prefrontal and posterior regions (Chavez-Eakle et al., 2007; de Souza et al., 2010). For instance, de Souza et al. (2010) used SPECT to examine 17 patients with behavioral variant of fronto-temporal lobar degeneration (bvFTD), and showed brain regions in which perfusion correlated with creativity performance on the TTCT. Several of the reported regions overlapped or were very close to the ones shown in the present meta-analysis, in particular in the left IFG (BA 47), the left posterior inferior and middle temporal gyri (BA 37), the left inferior parietal lobule (BA39/40), and the left precuneus (BA 23).

The shared creativity network evidenced here includes regions usually associated with cognitive rather than affective processing. This finding suggests that this set of brain regions supports cognitive processes rather than affective, conative, or motivational processes (Lubart, 2003). This does not imply that the latter processes are not involved in creative thinking. One should keep in mind that this review was specifically designed to investigate the cognitive aspects of creative thinking rather than affective factors.

The brain regions shared by creativity tasks appear to be predominantly distributed in the left hemisphere (Figure 1). When comparing the number of left and right foci reported in the reviewed studies, the number of left foci (*n* = 266) was significantly greater than the right (*n* = 173) ones [paired *t*-test: *t*_(33)_ = 3.43, *p* = 0.002]. This predominance of left co-localizations, which was also observed in previous studies (Arden et al., 2010; de Souza et al., 2010; Dietrich and Kanso, 2010), does not support the hypothesis of right dominance for creativity (Bowden and Jung-Beeman, 2003; Jung-Beeman et al., 2004; Friedman and Forster, 2005; Howard-Jones et al., 2005; Arden et al., 2010; Dietrich and Kanso, 2010). Furthermore, the left—but not right—dorsolateral PFC and left anterior temporal lobe have been shown to be critical for creativity tasks in brain stimulation studies (Cerruti and Schlaug, 2009; Chi and Snyder, 2011, 2012; Metuki et al., 2012). The leftward asymmetry observed in the present study is unlikely due to a domain effect, since both verbal and non-verbal stimuli were associated with a left-dominant network (60% of the foci were left-sided in both verbal and non-verbal experiments). Overall, available evidence shows that both hemispheres are involved in creative thinking, and it is possible that right regions are specialized for specific processes (see further discussion below in relation to the combination vs. generation comparison).

### Semantic and executive roles of the shared creativity network

The shared creativity regions evidenced by this meta-analysis include areas involved in semantic processing (Buckner et al., 2008; Binder et al., 2009; Price, 2010; Seghier et al., 2010; Vigneau et al., 2010): the IFG (BA47), the left posterior parietal lobule, and the left posterior part of the lateral middle temporal region. Some of these regions, namely the left IFG and posterior part of the left lateral temporal cortex, were more associated with verbal than non-verbal tasks in the subsequent analysis, reinforcing the hypothesis that these regions fulfil the semantic requirements of creativity tasks. The left IFG is indeed thought to play a crucial role in the controlled retrieval of information in semantic memory and/or in the selection of semantic associates in competition during retrieval (Thompson-Schill et al., 1997; Wagner et al., 2001; Thompson-Schill, 2003; Badre and Wagner, 2004, 2007; Kan and Thompson-Schill, 2004; Martin and Cheng, 2006; Thompson-Schill and Botvinick, 2006). According to its multimodal integrative functions and its role in semantic memory (Binder et al., 2009), the posterior parietal lobule (BA 39) may be essential to the integration of different types of semantic information. The lateral temporal cortex has been associated with the activation of semantic concepts and the integration of their meaning (Price, 2010).

The shared creativity network includes several prefrontal-parietal sub-regions. Parieto-prefrontal networks have also been found associated with fluid reasoning (e.g., the P-FIT theory from Jung and Haier, 2007) and executive functions, though their exact location is difficult to compare to the current results. Among the present prefrontal regions, overlaps were found between task-dependent maps in several discrete regions (overlap between combination and generation maps in Figure 2A, overlap between verbal and non-verbal tasks maps in Figure 3A). Both overlaps included a frontal region located in the caudal part of the IFG and the IFJ, extending to the adjacent middle frontal gyrus (BA 44, 45/47). This region has been associated with several executive processes, including cognitive control (Koechlin et al., 2003; Derrfuss et al., 2005; Azuar et al., 2010), inhibition, and flexibility (Miller and Tippett, 1996; Aron et al., 2003; Rieger et al., 2003; Derrfuss et al., 2005; Picton et al., 2007; Volle et al., 2012), fluency (Perret, 1974; Bates et al., 2003; Krainik et al., 2003; Hillis et al., 2004; Kinkingnehun et al., 2007), and working memory (Goldman-Rakic, 1987; Duncan and Owen, 2000; Mottaghy et al., 2002; Curtis and D'Esposito, 2003; Sakai and Passingham, 2003; Courtney, 2004; Volle et al., 2005, 2008; Mohr et al., 2006; Mottaghy, 2006; Postle, 2006; Sala and Courtney, 2007; Tsuchida and Fellows, 2009).

Although this meta-analgfysis was not designed to determine the specific executive processes supported by these regions, it is nevertheless interesting to consider their link with creativity tasks, as several theoretical frameworks rely on the involvement of the executive processes in creative thinking (Carlsson et al., 2000; Dietrich, 2004; Bogousslavsky, 2005; Changeux, 2005). Among these processes, fluency is critical for divergent thinking tasks, such as the TTCT. Chavez-Eakle et al. (2007) showed a region within the left IFG (BA 47, 11) in which CBF correlated with fluency performance on the TTCT in healthy subjects. More specifically, among the criteria measured by the TTCT, the inferior frontal region was related to the fluency (as well as flexibility) aspects of the task, whereas CBF in a more anterior region in the rostral PFC (BA 10) co-varied with the originality of responses. Cognitive flexibility (set shifting and task switching tasks) has been consistently associated with the IFJ, together with the posterior parietal cortex (Derrfuss et al., 2005; Kim et al., 2011). In contrast to classical set shifting or task switching paradigms, shifts in creativity tasks are not specified by an instruction or by feedback, but are initiated spontaneously by the individual. In relation to spontaneous flexibility, previous patient studies (Miller and Tippett, 1996; Goel and Grafman, 2000) have suggested a role for the right inferior frontal region in hypothesis generation with set-shift transformation—processes that may be necessary in most creativity tasks. The lateral PFC, and especially the IFJ and/or right IFG have also been associated with inhibition of prepotent but inappropriate responses and switching to an alternative response (Miller and Tippett, 1996; Garavan et al., 1999; Konishi et al., 1999; Liddle et al., 2001; Menon et al., 2001; Aron et al., 2003; Rieger et al., 2003; Brass et al., 2005; Derrfuss et al., 2005; Picton et al., 2007; Xue et al., 2008; Kenner et al., 2010; Walther et al., 2010; Volle et al., 2012).

Cognitive flexibility and inhibition of prepotent responses could be related to processes that enable thinking away from conventional or constrained ideas (Munakata et al., 2011), a fundamental principle of most creativity tasks, including divergent thinking and problem-solving tasks. In divergent thinking tasks, originality depends on the ability to provide unusual answers and may require the suppression of more obvious responses. In problem-solving tasks, problems are typically biased by constraints that are implicitly induced by the problem and that prevent participants from considering and evaluating the correct solutions (Knoblich et al., 1999; Frith, 2000; see also Reverberi et al., 2005; Chi and Snyder, 2011). Relaxing constraints in the semantic domain, for instance in a sentence completion task (Burgess and Shallice, 1997), also relies on the lateral PFC (Nathaniel-James and Frith, 2002). Further exploration is needed to determine whether thinking away from constraints and more classical executive functions, namely cognitive flexibility and inhibition, rely on similar lateral prefrontal regions (the IFG or middle frontal gyrus).

Overall, several regions—especially in the lateral PFC—may support the semantic and executive processes involved in various creativity tasks. These processes may participate in knowledge activation and its control, enabling ideation (Table 9).

**Table 9:** **Summary table of the results and hypothetical roles of shared and task-oriented creativity regions**.

**Regions**	**Hypothetical roles**
**SHARED REGIONS: MAY REFLECT COGNITIVE CONTROL AND SEMANTIC MEMORY REQUIRED FOR IDEATION**	
left IFJ (BA44/6) extension to DLPFC	Flexible cognitive control on information retrieved from memory and activation of task representations (Brass et al., 2005)
	In interaction with dorsal ACC (BA 32) (Beckmann et al., 2009)
Left IFG (BA45/ 47)	Controlled retrieval and/or selection of remote information from semantic memory (Martin and Cheng, 2006; Thompson-Schill and Botvinick, 2006)
	May control retrieval in connected parietal systems (BA 39) and allow higher levels of abstraction (Binder et al., 2009)
Left GA (BA 39)	Concept retrieval from episodic and semantic memory, integration of different types of semantic information (Binder et al., 2009; Bonner et al., 2013)
**COMBINATION ORIENTED: MAY BE INVOLVED IN RELATIONAL REASONING AND ABSTRACT THINKING**	
Left RPFC (BA 10/47 and 46)	Relational integration of concepts or mindsets (Christoff et al., 2001, 2003; Bunge et al., 2005)
	Internal-generation of an integrated abstract mindset (Christoff et al., 2009a)
	Monitoring of tasks and subtasks (Koechlin et al., 1999) engaged in combination
Left posterior MTG (BA 37/21)	Storage, activation or retrieval of perceptual information about objects and their attributes, rules and concepts, integration of their meaning (Binder et al., 2009; Price, 2010)
Right IFG (BA 44/45)	Suppression of inappropriate mindsets or responses (Aron et al., 2003; Volle et al., 2012) and switching to alternatives
	Lateral transformation of the problem (Goel and Vartanian, 2005)
**UNUSUAL GENERATION ORIENTED: MAY BE RELATED TO INCREASED WORKING MEMORY REQUIREMENTS**	
Left DLPFC (BA 45/46)	Updating and manipulation of mindsets in working memory (verbal/semantic content)
	Free selection of responses in working memory (Rowe et al., 2008)
Left SMG (BA 40)	Maintenance on mindsets in working memory (Smith and Jonides, 1999)
Medial rostral PFC (BA10)^*^	Evocation of unusual semantic associates (Shamay-Tsoory et al., 2011; Green et al., 2012) in an associative mode of activation of mental representations

* This region was found to be associated with unusual generation tasks, but was not significant when contrasting unusual generation with combination tasks.

### Specializations of different regions for distinct task demands

#### Regions showing greater activity for combination than unusual generation tasks (Table 9)

This meta-analysis also suggests that specific brain regions may support specific creative tasks, with combination and generation tasks activating partly non-overlapping brain regions (Figure 2). The rostral portion of the PFC (BA 10) was particularly sensitive to this distinction. Statistical comparison between task categories (Figure 2B) showed that combination tasks, more than the other task types, recruited the lateral rostral PFC together with the posterior lateral temporal and temporo-parietal regions. The lateral rostral PFC is generally activated by tasks that require integration of multiple relations (Christoff et al., 2001; Kroger et al., 2002; Smith et al., 2007), analogical reasoning, and similarity judgment (Wharton et al., 2000; Bunge et al., 2005; Geake and Hansen, 2005; Green et al., 2006; Wendelken et al., 2008; Crone et al., 2009; Garcin et al., 2012; Vartanian, 2012), abstract thinking (Badre, 2008; Christoff et al., 2009a, 2001) as well as coordinating goals and sub-goals (Koechlin et al., 1999; Koechlin and Hyafil, 2007). All of these functions may be involved in combination tasks. Therefore, the lateral rostral PFC could play a role in enabling subjects to find combinatorial solutions based on remote semantic associations or on relational similarity. This hypothesis is consistent with recent models that place the lateral rostral PFC at the top of a hierarchical organization of prefrontal functions according to progressively higher levels of abstraction (Christoff et al., 2001; Hampshire et al., 2010; Krawczyk et al., 2010), or to a greater distance from external stimuli when building internal thoughts (Christoff et al., 2003; Burgess et al., 2007). More posterior areas of the PFC are thought to be involved in the systematic control of representations necessary for these higher-level processes (Kroger et al., 2002; Brass et al., 2005; Cho et al., 2010; Wendelken and Bunge, 2010), and may be sufficient in some creativity tasks, such as free generation tasks. The rostral PFC is likely to operate as part of a network, together with other regions, such as the posterior STG and the temporo-parietal junction (BA 39), as suggested by the combination map and its contrast to the generation map. Further clarification is needed to determine the respective role of each region in detecting similarities and combining different elements. Anatomically, these co-activations may be supported by direct connections between the PFC and superior lateral temporal areas, as shown in monkeys by Petrides and Pandya (2007).

A right-lateralized IFG activation was more prominent for combination than for unusual generation. This finding may be related to the fact that tasks classified in the combination category included insight problem-solving tasks, which have been shown to involve the right IFG in relation to shifts in hypothesis generation (Goel and Vartanian, 2005). The right IFG is also associated with suppression of inappropriate mindsets or responses (Aron et al., 2003; Volle et al., 2012), which may be more important in combination than in unusual generation tasks, in order to suppress unsuitable self-generated responses. Insight in problem solving has also been associated with the right temporal pole, a region closely connected with IFG through the uncinate fasciculus (Jung-Beeman et al., 2004; Bowden et al., 2005). That this result rather reflects a stronger interaction between the two hemispheres in order to combine ideas cannot be ruled out (Takeuchi et al., 2010b).

#### Regions showing greater activity for unusual generation than combination tasks (Table 9)

While combination tasks engaged the lateral rostral PFC, unusual generation maps showed the involvement of its medial part (Figure 2A). Although this rostromedial PFC region was not significant when contrasting unusual generation to combination maps (Figure 2B), this result is in agreement with a lesion study that showed that the medial rostral PFC region is critical for unusual generation performance (Alternate Uses tasks and TTCT) and, more specifically, that it is associated with the unusualness (originality) of the responses (Shamay-Tsoory et al., 2011). The role of the medial rostral PFC (BA 10) may not be limited to the evocation of unusual responses in generation tasks. Green et al. (2012) found more activation in this region when the domains compared in analogical reasoning were remote. Thus, semantic distance or information dissimilarity might be coded in this region. This may explain that activation in the rostromedial PFC was not statistically significant when comparing directly unusual generation to combination tasks (Figure 2B), since the semantic distance/dissimilarity factor may have an effect on both. It is noteworthy that the link between the medial rostral PFC and novelty/unusualness has been made outside the scope of creativity studies. For example, Krueger et al. (2007) showed scripts of real life events to subjects participating in an fMRI experiment and found that the medial rostral PFC was involved in coping with unusual situations more than with frequent ones. This region has also been associated with counterfactual thinking (Gomez Beldarrain et al., 2005; Van Hoeck et al., 2013), prospective memory and future thinking (Hassabis et al., 2007; Schacter et al., 2007; Abraham et al., 2008; Addis et al., 2009; Szpunar et al., 2009; Burgess et al., 2011; Volle et al., 2011), mentalizing (Frith and Frith, 2006; Andrews-Hanna et al., 2010; Gilbert et al., 2010), and daydreaming (Christoff et al., 2009b; Mason et al., 2007). These cognitive functions may be involved in the search for alternative responses in generation tasks. The relative role of medial vs. lateral rostral PFC in creative thinking will be an interesting question to address in future studies, in terms of representations or processes, and in the light of existing theories (Burgess et al., 2007; Buckner et al., 2008; Christoff et al., 2009b).

Unusual generation tasks, when compared statistically to combination tasks (Figure 2B), were associated with the dorsolateral prefrontal area (BA 9), the anterior inferior parietal region (left inferior parietal lobule and dorsal supramarginal gyrus, BA 40), and the cerebellar lobes (Figure 2B). This result is consistent with a previous finding from Chavez-Eakle et al. (2007) based on correlations between CBF and performance on TTCT. The supramarginal gyrus and middle frontal gyrus are anatomically connected (Catani and Thiebaut de Schotten, 2012). The involvement of this fronto-parietal network in generation tasks could be related to its role in working memory, monitoring, and/or attention- a set of functions more involved in generation than in combination tasks. The fronto-parietal regions have also been associated with the free and/or random generation of actions (Frith et al., 1991; Rowe et al., 2008) that requires the freedom of choice of one's responses as well as their selection and monitoring. Both are likely to participate in creative generation tasks that engage spontaneous willed actions. The role of the cerebellum in human cognition is poorly understood. A recent meta-analysis of functional imaging studies showed the involvement of the cerebellar lobes in six high-level functions: emotion, working memory, executive functions, music, timing, and language (E et al., 2012) All these domains may be involved in creativity tasks, and it is difficult to draw precise conclusions from the present study. Nevertheless, cerebellar lobes appeared to be more associated with verbal than non-verbal tasks in the present meta-analysis, which is in agreement with the particular involvement of lobule VI (Declive) in language reported by E et al. (2012).

#### Verbal vs. non-verbal experiments

The separated ALE analyses for verbal and non-verbal experiments (Figure 3A) and the statistical comparison between them (Figure 3B) showed that tasks using verbal material engage more inferior regions than those using non-verbal ones in the lateral prefrontal, occipital, and medial frontal regions. A ventral/dorsal dissociation according to verbal/non-verbal domain of information in the caudal part of the lateral PFC has often been reported (Goldman-Rakic, 1987; Mottaghy et al., 2002; Curtis and D'Esposito, 2003; Sakai and Passingham, 2003; Courtney, 2004; Mottaghy, 2006; Mohr et al., 2006; Postle, 2006; Sala and Courtney, 2007; Volle et al., 2008). The analysis of non-verbal tasks showed bilateral activation in dorsal prefrontal areas (superior frontal gyrus), in regions implicated in attention, visuospatial processing, and working memory. Compared to verbal tasks, non-verbal tasks were also associated with more activation foci within the left rostral PFC, in the left occipital cortex, and in bilateral dorso-caudal prefrontal regions. However, because the non-verbal experiments gathered heterogeneous stimuli (visual, spatial, music), it is difficult to interpret these results and their apparent left dominance.

Finally, this second set of results supports the view that distinct creativity tasks could make different demands on cognitive processes that are subserved by different brain regions, in particular in rostral vs. caudal prefrontal areas. These regions, when damaged, may affect some aspects of creativity but each in a different manner.

## Conclusion

The present findings highlighted the importance of caudal and rostral prefrontal regions, together with inferior parietal and posterior temporal areas, for the cognitive aspects of creativity. We further showed that some of these regions (mainly prefrontal ones) were shared by all task categories investigated, whereas other regions were more specifically associated with particular tasks. The core creativity network outlined by this meta-analysis is consistent with previous findings from different approaches in both healthy subjects and patients. Within this network, the lateral PFC (and especially the left IFJ) has been associated with various executive processes, such as fluency, flexibility, inhibition of prepotent responses, and cognitive control. These processes may represent components of creative thinking. In addition, this core network includes semantic regions, i.e., the left angular gyrus, STG and IFG, which have been related to the retrieval or connection of semantic associates. Retrieving and activating distant mental representations may constitute some of the mechanisms that allow creativity to emerge in both combination and free generation tasks. Subsequent task-dependent analyses revealed more specific regions in rostral PFC and in parieto-temporal regions. Among them, the lateral rostral PFC and posterior temporal regions, associated with combination tasks, may more specifically support the ability to combine information in new ways, bridging semantic distances and/or superficial dissimilarities between them. A more caudal dorsolateral PFC region together with the inferior parietal lobule, associated with generation tasks, might rather support the free production of unusual or alternative responses. However, the cognitive processes involved in creativity are not yet understood, and their identification was outside the scope of this study. This meta-analysis does not enable us to determine whether or not the observed regions support processes specific to creative thinking. Further studies should explore whether and how original ideas emerge automatically from remote activation in semantic networks or whether they result from an effortful cognitive set of processes.

### Conflict of interest statement

The authors declare that the research was conducted in the absence of any commercial or financial relationships that could be construed as a potential conflict of interest.
